# Synergistic Enhancement of the High-Temperature Friction and Wear Behavior of PTFE Matrix Composites with the Addition of CF, PEEK, and TiC

**DOI:** 10.3390/polym16233412

**Published:** 2024-12-04

**Authors:** Lin Yuan, Yunxiang Han, Jinming Zhen, Zhengfeng Jia, Ran Zhang

**Affiliations:** College of Materials Science and Engineering, Liaocheng University, Liaocheng 252059, China; 19111912571@163.com (L.Y.); jiazhengfeng@lcu.edu.cn (Z.J.); zhangranlicp@163.com (R.Z.)

**Keywords:** PTFE matrix composite, PEEK, dry sliding, high temperature

## Abstract

With the rapid development of the aerospace, automobile, and ocean industries, there is an urgent need for the fabrication of high-performance polymer matrix composites with low friction and wear in wide temperature ranges. In this paper, polytetrafluoroethylenes (PTFEs) doped with polyether-ether-ketone (PEEK), carbon fiber (CF), and TiC were prepared, and the effects of testing temperatures from room temperature to 250 °C in air conditions were investigated. The results showed that the friction coefficient of the PTFE matrix composites had no obviously changing trend, while the wear resistance properties were significantly improved. Due to the synergistic lubrication and enhancement of CF, PEEK, and TiC, the wear rate for composites with these particles decreased from (2.04–2.72) × 10^−3^ mm^3^/Nm for pure PTFE to (0.67–1.96) × 10^−4^ mm^3^/Nm. Moreover, the SEM analysis results showed that the main wear mechanisms are fatigue and abrasive wear for the PTFE matrix composites. The results obtained in this study will provide data and technical support for the development of high-performance polymer matrix composites with low friction and wear that can be used over a wide temperature range.

## 1. Introduction

Due to its excellent resistance to corrosion and high temperatures and its self-lubricating properties, polytetrafluoroethylene (PTFE) has been widely used as a solid lubricating material in various industries, like aerospace, automobiles, medical devices, etc. [1,2,3,4,5,6,7]. However, its poor wear resistance limits its application in a wider range of fields, like those under high load/sliding speeds and wide temperature ranges, etc. [8,9,10]. Thus, it is quite important to improve the mechanical and tribological properties of PTFE matrix composites and broaden their applications under complex/severe environments.

Up to now, many studies have been performed by researchers to increase the frictional performance of PTFE matrix composites [4,11,12,13,14,15]. Among these studies, incorporating second reinforced particles is the main effective way to increase the wear resistance properties of PTFE matrix composites. With the aim of improving the wear resistance of PTFE matrix composites, Sun et al. investigated the effect of soft-phase PEEK and hard-phase ZrO_2_ on the tribological performance of PTFE materials. Their results showed that soft and hard fillers could inhibit interlayer slippage within the molecular chains and decrease the wear rate for composites [16]. Xue et al. fabricated and investigated the mechanical/frictional performance of a series of Ti_3_C_2_T_x_ MXene-reinforced PEEK-PTFE composites and found that due to the strong bonding between MXene and PEEK/PTFE, the compressive strength was improved by 28.37%, and the wear rate reduced by 40% [8]. Using the DC electric-field-assisted hot-pressing sintering method, Liu et al. prepared and studied the effect of the Mg–Al-layered bimetallic hydroxide (LDH) proportion on the frictional and wear behavior of polyamide–imide (PAI)/PTFE composites; the results showed that when the Mg–Al LDH content was 3%, the PAI composite film had the best lubricating and wear resistance properties [17]. Raut et al. investigated various reinforced fillers (carbon black, Gr, and bronze) on the tribological performance of polytetrafluoroethylene (PTFE) matrix composites under a fixed load (32 N) and sliding speed (0.5 m/s) for 1 h in dry sliding conditions. The results showed that PTFE matrix composites reinforced with the aromatic thermosetting copolyester exhibited higher wear resistance and a lower coefficient of friction compared to carbon black, bronze, and graphite fillers [18]. Based on the representative volume element model, He et al. investigated the effect ratios of 0.5 mm short carbon fibers (SSCFs) and 400-mesh short carbon fibers (LSCFs) on the mechanical and tribological properties of PTFE matrix composites. The results showed that the composite with 5 wt. % LSCFs/10 wt. % SSCFs presented the best overall performance compared to the addition of LSCFs and SSCFs alone [19].

Furthermore, it is also important to investigate the surrounding environment’s effect on the friction and wear behavior of the composite, as the frictional process is complex [2,20,21,22,23,24,25,26]. With the aim of investigating the effect of trace moisture in hydrogen gas on the frictional behavior of PTFE materials, Chen et al. studied the tribological behavior of CF-filled PTFE composites under this condition; the results showed that the coefficient of friction increased first and then decreased with an increase in water content, but the wear rate gradually increased with an increase in moisture [27]. Amenta et al. studied the frictional behavior of PTFE matrix composites (reinforced by glass fiber (GF) and carbon fiber (CF)) coupled with AISI 304 stainless steel (with and without sprayed Cr_2_O_3_). The results showed that the GF-reinforced PTFE composites had the lowest friction coefficient against both counterfaces [28]. Through incorporating polyaryl ether sulfone/polymethyphenlsiloxane microcapsules and carbon fibers into PTFE/aramid fabrics, Lu et al. studied the friction and wear of these composites under high temperatures and heavy loading. The results showed that the incorporation of microcapsules and carbon fiber multilayer structures could improve the tribological properties of the composites (the friction coefficient and wear rate were reduced by 17% and 24%) under severe conditions [29]. Xu et al. coated Nomex/PTFE fabric composites with PTFE/MoS_2_ coatings and investigated the tribological properties over a wide temperature range (−150–25 °C); the results showed that with a decrease in testing temperature, the wear rate for the composite decreased, and it exhibited unmeasurable wear at −150 °C [30].

As shown in the literature, the frictional behavior of PTFE matrix composites/coatings is quite dependent on the composition content and surrounding environment. From the above summary of the literature, we can see that there are fewer studies that systematically investigate the friction and wear behavior of PTFE matrix composites over a wide temperature range in recent years, and the wear mechanisms at high temperatures are complex. In this paper, with the aims of further improving the wear resistance properties of PTFE matrix composites and studying the effect of testing conditions on their frictional performance, PTFE matrixes with carbon fiber (CF), PEEK, and TiC ceramic particles were fabricated, and the synergistic enhancement effects were investigated from room temperature to 250 °C. Moreover, the related wear mechanism was discussed in detail.

## 2. Experimental Details

### 2.1. Material Preparation

The PTFE matrix composite was fabricated using the cold and hot sintering method. Firstly, the powders of PTFE, CF, TiC, and PEEK were thoroughly mixed in absolute ethanol, and then, the mixed powder was dried in an oven under a temperature of 60 °C for 6 h. After that, the dried mixed powder was placed into a stainless steel mold for cold pressing and shaping. Finally, the material was subjected to sintering in a furnace at 350 °C for 2 h to obtain the desired PTFE matrix composites. The composition of the PTFE matrix composites is listed in Table 1. The experimental raw materials and instruments used in this study are as follows: PTFE (Liaocheng Flore New Material Technology Co., Ltd., Liaocheng, China, medium particle ML0 type); PEEK (UK Vickers 450 G injection molded plastic particles, 500 mesh); TiC (Shanghai Naiou Nanotechnology Co., Ltd., Shanghai, China, 300 mesh).

### 2.2. Tribological Tests

**Figure 1:**
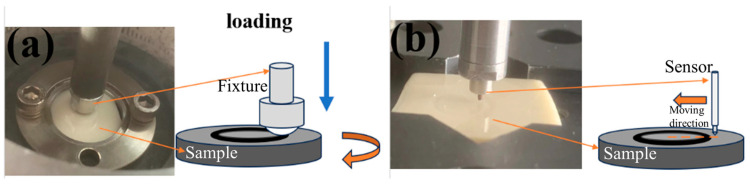
Schematic diagram of friction (**a**) and wear rate (**b**) testing machine.

Tribological tests were performed on an HT-1000 ball-on-disk high-temperature tribometer (Lanzhou Zhongke Kaihua Technology Development Co., Ltd., Lanzhou, China) in rotation mode, as shown in Figure 1a. The tribotests were run in air, sliding against GCr15 bearing steel (6.0 mm, 700HV1), under the following conditions: a sliding speed of 0.33 m/s, a duration of 30 min, a normal load of 5 N and a wear track diameter of 10 mm. The testing temperatures were selected as 25, 50, 100, 150, 200 and 250 °C, respectively. During the sliding process, the coefficient of friction (COF) for tribopairs was recorded automatically by a computer. The volume of the wear track for composites after the tribotest was measured by a contact surface profilometer (MT-500, Lanzhou Zhongke Kaihua Technology Development Co., Ltd., Lanzhou, China), as shown in Figure 1b, then the wear volume was divided by the load (N) and sliding distance (m), with the unit expressed as mm^3^N^−1^m^−1^. To decrease the accuracy of the tribological test, the measurement was performed at least three times under the same testing conditions.

### 2.3. Characterization

For the sintered samples, the phase composition of the PTFE matrix composites was characterized by X-ray diffraction (XRD, TD-3700, 40 kV, 30 mA, CuKα,) with a range of 5–80° under the θ–2θ scan mode. To analyze the wear mechanism of the PTFE matrix composites, the worn surface characteristics of the worn surface were investigated by scanning electron microscopy (SEM, JSM 5600LV, Zeiss, Germany).

## 3. Results and Discussion

**Figure 2:**
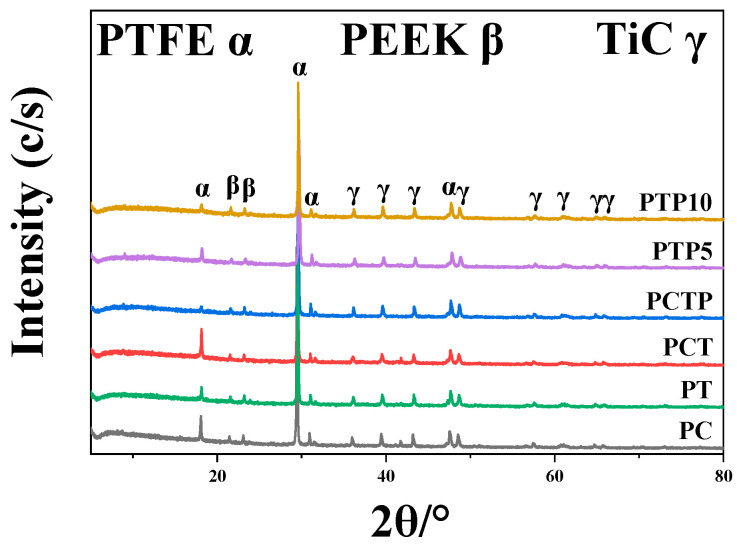
XRD pattern of the PTFE matrix composite.

Phase compositions of the six PTFE matrix composites will be analyzed by the XRD pattern, as shown in Figure 2. It can be seen that the composites are composed of three different phases: the PTFE matrix (three diffraction peaks at 2θ of 18.2°, 29.6°, and 47.7°), PEEK (three diffraction peaks at 2θ of 18.7°, 20.7°, and 22.8°) and TiC. In addition, it can be found that the relative strength of PTFE peak decreases with the increase in PEEK powder and TiC ceramic particles.

**Figure 3:**
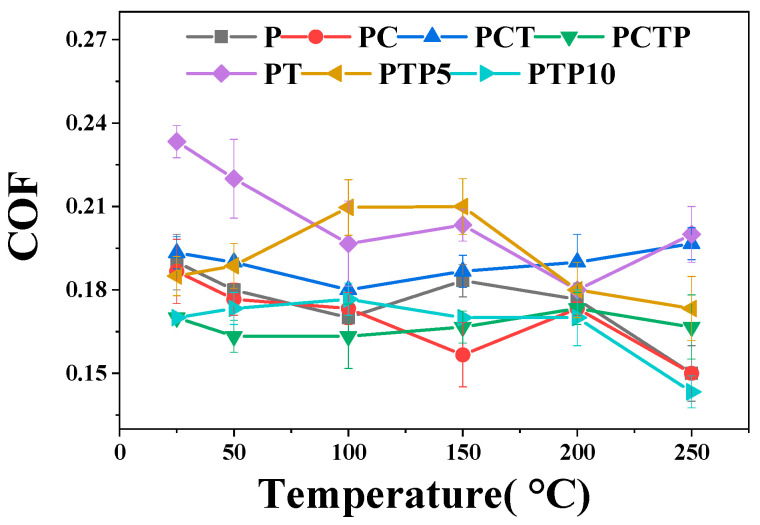
Friction coefficient of the PTFE matrix composites as a function of testing temperature.

Figure 3 exhibits the changing trend of COF for these six PTFE matrix composites at various testing temperatures from 25 to 250 °C. It can be clearly see that the COFs vs. temperatures change similarly for composites of PCT, P, PCTP and PTP10: as the testing temperature increases from 25 to 200 °C, COFs of these composites show a slight fluctuation (P: 0.17–0.2; PCT: 0.18–0.19; PCTP: 0.16–0.17; PTP10: 0.17–0.18), while at 250 °C, due to the softening of the worn surface and the increased diffusion rate of the soft phase (PTFE and PEEK), the COFs decrease to the minimum for P (0.15), PTP10 (0.14) and PCTP (0.17) composites. As for the PCT material, it remains at 0.19. For the PT composite, when the testing temperature increases from 25 to 200 °C, the COFs show a slightly increasing (0.23 to 0.24) trend; as the surrounding temperature increases to 200 °C, the value decreases to the minimum of 0.18, then it increases slightly at 250 °C (0.20). For the PC composite, the COFs’ change trend is different; as the testing temperature increases from 25 to 150 °C, it continuously decreases to a low value of 0.16, then the COF increases slightly at 200 °C, while at 250 °C, the COF decreases to the minimum of 0.15. As for the PTP5 composite, the COF continuously increases to the maximum of 0.21 from 25 to 150 °C, then as the testing temperature increases to 250 °C, the best lubricating properties are observed (COF:0.17). For the effect of composition, we can see that from 25 to 150 °C, composites of PTP10, PCTP, PC and pure PTFE have similar COFs, which range from 0.16 to 0.20, while the values are high for PT and PTP5 composites (0.19–0.24). At 200 °C, the COFs are around 0.18 for all six composites. The P, PTP10 and PC composites exhibit the lowest COF of 0.15 at 250 °C, while composites of PCT and PT show the highest value of about 0.20 at this temperature.

**Figure 4:**
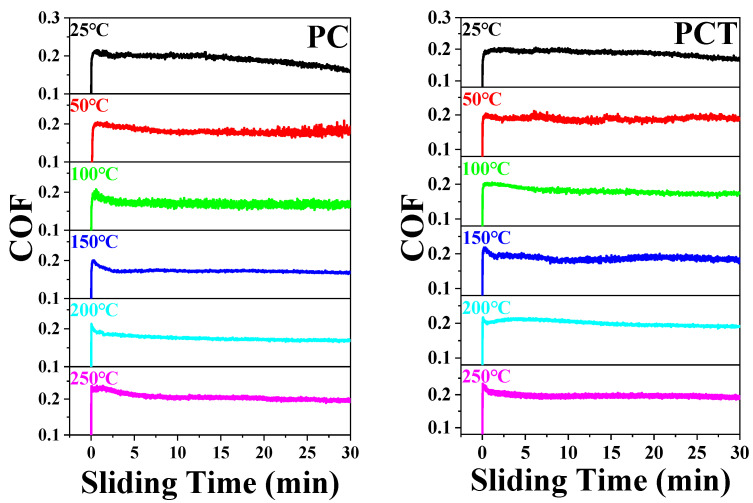

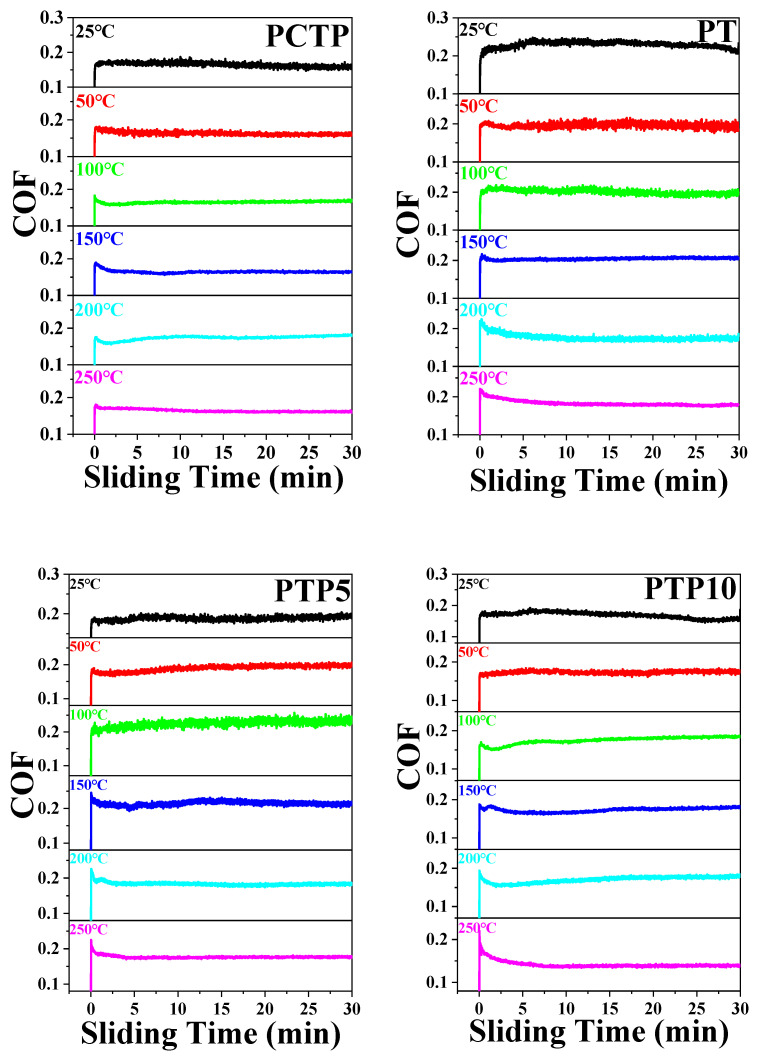
Typical friction curve of PTFE matrix composites at different testing temperatures.

Figure 4 presents the typical COFs vs. sliding time curves of the PTFE matrix composites at various testing temperatures from 25 to 250 °C. It can be seen that the changing trends of COFs with the sliding time are basically the same for these composites: in the running stage, the fluctuation om COF (within 5 min) is large and in the stable stage, the value become smooth, especially for the composites of PCT and PCTP, indicating that tribofilm has formed on the worn surface. Moreover, we can also see that at high temperatures (100–250 °C), the running stage becomes short, and in the stable stage, the COF fluctuation is smaller than that at low temperatures (25–100 °C).

**Figure 5:**
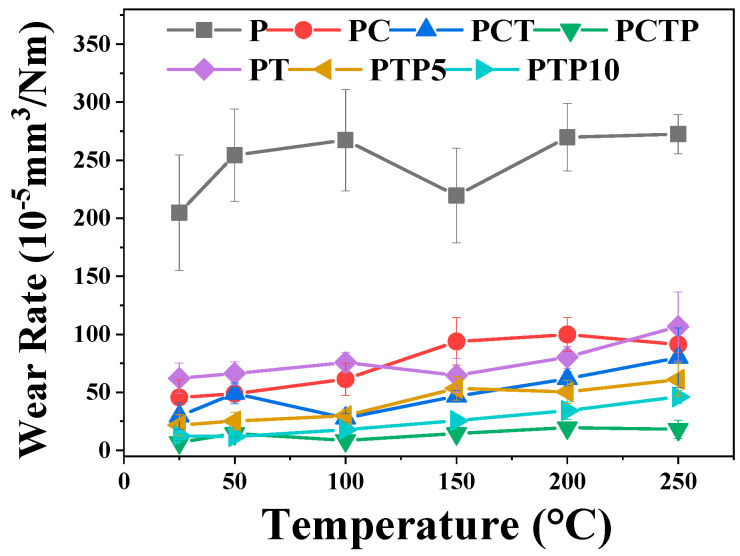
Wear rate of the PTFE matrix composites at different sliding temperatures.

Figure 5 shows the wear rate of the PTFE matrix composites coupled with a GCr15 stainless steel ball under various testing temperatures. All six composites exhibit relatively lower wear rates as compared with that of the pure PTFE sample; the value decreases from (2.04–2.72) × 10^−3^ mm^3^/Nm for pure PTFE to (0.674–10.6) × 10^−4^ mm^3^/Nm for other composites, especially for PCTP, the value for this being below 1.96 × 10^−4^ mm^3^/Nm. The results indicate that the addition of a hard phase (TiC), fiber (CF) and a soft phase (PEEK) have a synergistically enhancing effect, decreasing the wear rate of the PTFE matrix composite. For the effect of the testing temperature, the wear rate exhibits an increasing trend for these PTFE matrix composites with the increase in the testing temperature from 25 to 250 °C. For the composite of PT, the wear rate increases slightly from 25 to 100 °C (6.2–7.6 × 10^−4^ mm^3^/Nm), then it decreases to 6.4 × 10^−4^ mm^3^/Nm at 150 °C; as the testing temperature increases to 250 °C, composite exhibits the largest wear rate (10.7 × 10^−4^ mm^3^/Nm). The PC, PCTP and PTP10 composites show similar changing trends: the wear rate gradually increases to the maximum value from 25 to 200 °C for the PC and PCTP (PC: 9.97 × 10^−4^ mm^3^/Nm and PCTP: 1.96 × 10^−4^ mm^3^/Nm) and 250 °C for the PTP10 (4.6 × 10^−4^ mm^3^/Nm) composites; this may be due to the reduced mechanical properties at high temperatures. For the PCT composite, the changing trend is different: from 25 to 50 °C, the value increases to 4.6 × 10^−4^ mm^3^/Nm, and at 100 °C, it decreases to 2.78 × 10^−4^ mm^3^/Nm, then the wear rate increases slightly to the maximum value of 8.0 × 10^−4^ mm^3^/Nm at 250 °C. For the PTP5 composite, as the testing temperature increases from 25 to 150 °C, the wear rate continuously increases to 5.34 × 10^−4^ mm^3^/Nm, then it fluctuates within (5.04–6.11) × 10^−4^ mm^3^/Nm as the testing temperature increases to 250 °C.

**Figure 6:**
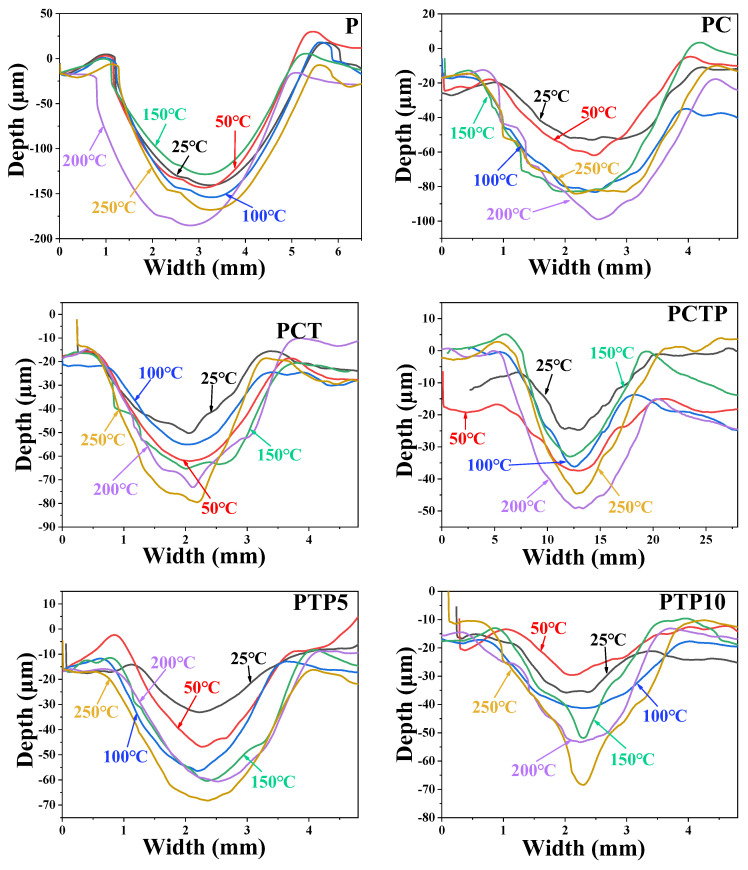
Cross-section of the worn surfaces for PTFE matrix composites at various testing temperatures.

**Figure 7:**
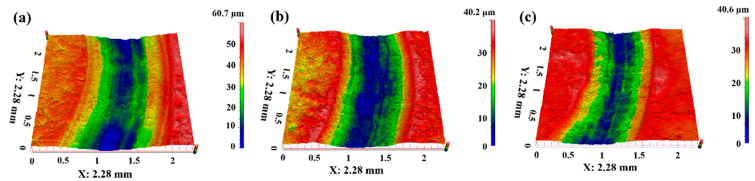

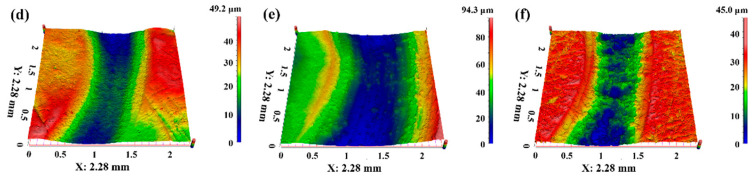
Three-dimensional morphologies of the worn surfaces for PTFE matrix composites at different testing temperatures: (**a**) PC-25 °C, (**b**) PCT-25 °C, (**c**) PCTP-25 °C, (**d**) PT-25 °C, (**e**) PTP5-25 °C, (**f**) PTP10-25 °C.

**Figure 8:**
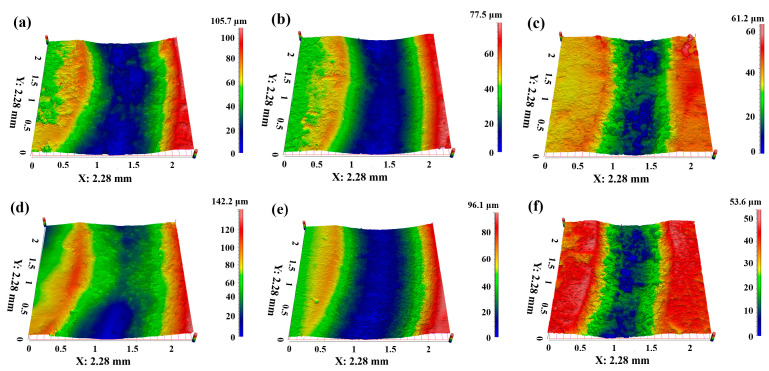
Three-dimensional morphologies of the worn surfaces for PTFE matrix composites at different testing temperatures: (**a**) PC-150 °C, (**b**) PCT-150 °C, (**c**) PCTP-150 °C, (**d**) PT-150 °C, (**e**) PTP5-150 °C, (**f**) PTP10-150 °C.

**Figure 9:**
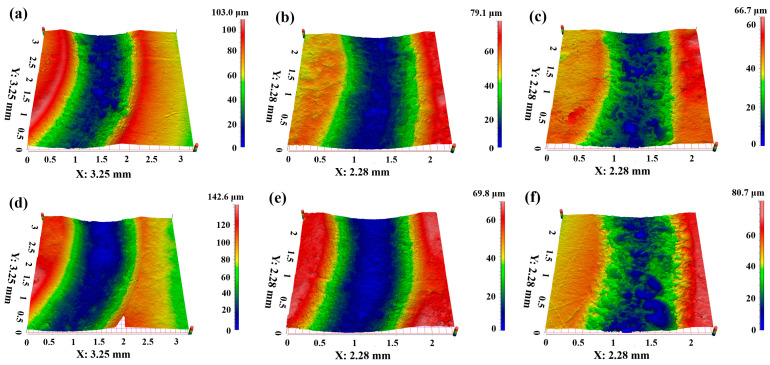
Three-dimensional morphologies of the worn surfaces for PTFE matrix composites at different testing temperatures: (**a**) PC-250 °C, (**b**) PCT-250 °C, (**c**) PCTP-250 °C, (**d**) PT-250 °C, (**e**) PTP5-250 °C, (**f**) PTP10-250 °C.

The wear resistance performance for PTFE matrix composites can also be reflected by the cross-section and three-dimensional morphology of the worn surface, as shown in Figure 6, Figure 7, Figure 8 and Figure 9. Figure 6 presents the cross-section of the worn surface for the PTFE matrix composite after sliding against the GCr15 stainless steel ball at various testing temperatures. It can be seen that the wear track is deep (100–200 μm) and broad (about 4 mm) for the pure PTFE sample. While as the soft phase, PEEK, hard phase, TiC, and CF are added, the wear track becomes narrow and shallow, the especially for PCTP composite, the depth is less than 50 μm, indicating that the wear-resistant layer is formed on the worn surface, and this is consistent with the changing trend of the wear rate (Figure 5). Figure 7, Figure 8 and Figure 9 illustrate the three-dimensional morphologies of the worn surface for PTFE matrix composites at 25, 150 and 250 °C. At 25 °C, the wear track is characterized by shallow grooves for composites of PC (Figure 7a), PCT (Figure 7b) and PCTP (Figure 7c), and they are smooth for PT (Figure 7d) and PTP5 (Figure 7e) samples. For PTP10, there are some delaminated pits on the worn surface (Figure 7f). As the testing temperature increases to 150 °C, the wear track becomes larger and deeper for almost all PTFE matrix composites as compared with that at 25 °C (Figure 8). Moreover, the worn surface becomes smooth and there are almost no grooves, and this may be attributed to the softening of the matrix; at 250 °C (Figure 9), the worn surface characteristics are similar to those at 150 °C.

**Figure 10:**
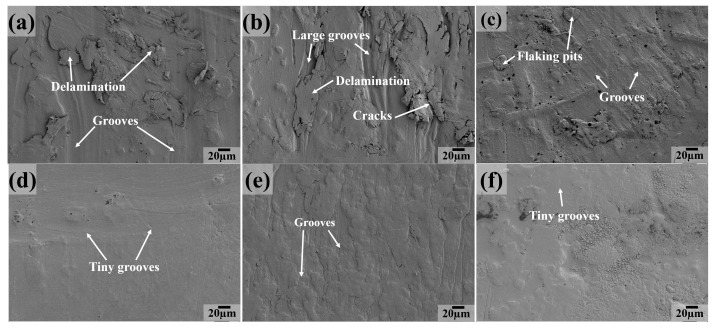
SEM images of the worn surfaces for PTFE matrix composites at 25 °C: (**a**) PC, (**b**) PCT, (**c**) PCTP, (**d**) PT, (**e**) PTP5, (**f**) PTP10.

**Figure 11:**
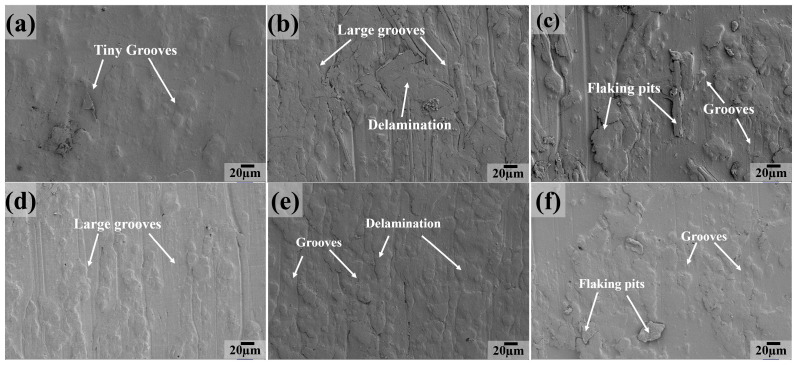
SEM images of the sliding surfaces for PTFE matrix composites at 50 °C: (**a**) PC, (**b**) PCT, (**c**) PCTP, (**d**) PT, (**e**) PTP5, (**f**) PTP10.

**Figure 12:**
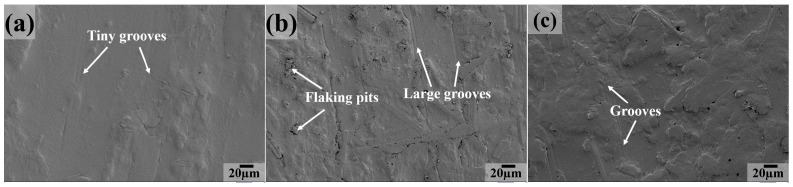

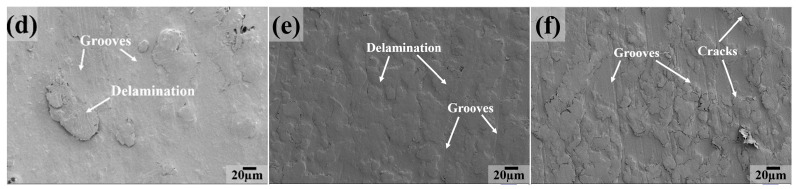
SEM images of the sliding surfaces for PTFE matrix composites at 100 °C: (**a**) PC, (**b**) PCT, (**c**) PCTP, (**d**) PT, (**e**) PTP5, (**f**) PTP10.

**Figure 13:**
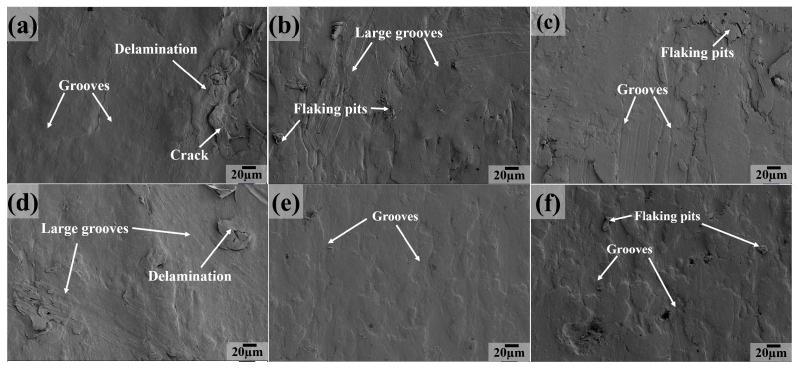
SEM images of the sliding surfaces for PTFE matrix composites at 150 °C: (**a**) PC, (**b**) PCT, (**c**) PCTP, (**d**) PT, (**e**) PTP5, (**f**) PTP10.

**Figure 14:**
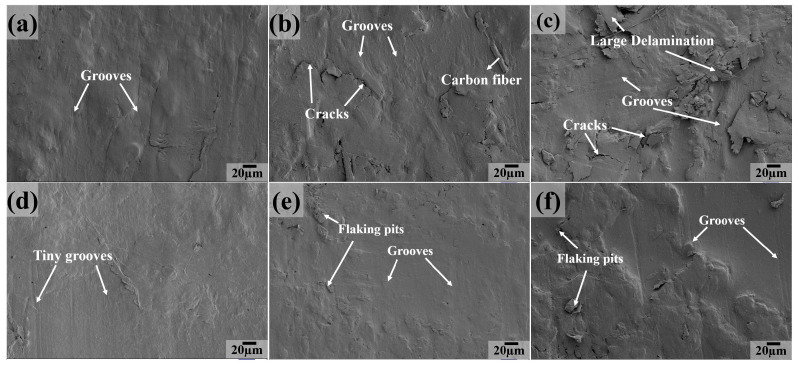
SEM images of the sliding surfaces for PTFE matrix composites at 200 °C: (**a**) PC, (**b**) PCT, (**c**) PCTP, (**d**) PT, (**e**) PTP5, (**f**) PTP10.

**Figure 15:**
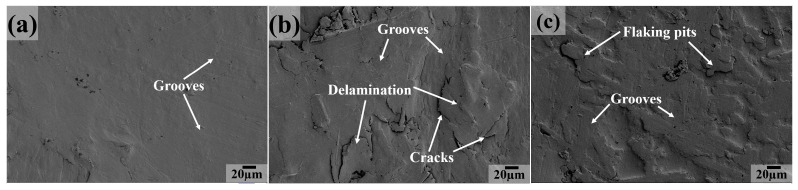

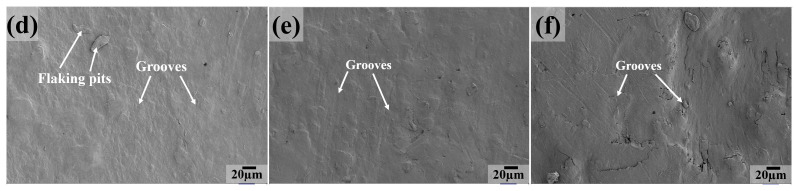
SEM images of the sliding surfaces for PTFE matrix composites at 250 °C: (**a**) PC, (**b**) PCT, (**c**) PCTP, (**d**) PT, (**e**) PTP5, (**f**) PTP10.

The SEM image was investigated to analyze the wear mechanism for PTFE matrix composites, as shown in Figure 10, Figure 11, Figure 12, Figure 13, Figure 14 and Figure 15. At 25 °C, a great extent of delamination and large grooves appear on the worn surface of the PC composite (Figure 10a), indicating that severe fatigue and abrasive wear are the main wear mechanisms. For the PCT sample, the worn surface characteristics are similar to those of the PC sample, both covered with grooves and delamination (Figure 10b). As for the PCTP and PTP5 composites, the extensive delamination and cracks disappear and only some flaking pits and grooves cover the worn surface (Figure 10c,e), suggesting that abrasive wear is the main wear mechanism. For PT and PTP10 composites (Figure 10d,f), the worn surface becomes smooth and there are no large grooves.

Figure 11 presents the worn surface as the testing temperature is 50 °C for PTFE matrix composites. The worn surface becomes smooth and there are only some tiny furrows (Figure 11a) for the PC composite as compared to the case at 25 °C, indicating that abrasive wear is the main wear mechanism. For the PCT, PCTP and PTP5 composites (Figure 11b,c,e), the worn surface characteristics are similar to those at room temperature. Large grooves instead of tiny grooves become the main feature for the PT composite (Figure 11d), suggesting that the main wear mechanism is abrasive wear. For the PTP10 (Figure 11f) composite, the worn surface is characterized by flaking pits and grooves.

Figure 12 shows the worn surface characteristics of six composites at a testing temperature of 100 °C. We can clearly see that the worn surface exhibits a similar morphology for PC, PCT and PTP5 composites (Figure 12a,b,e), compared with that at 50 °C. On the other hand, for the composite of PCTP (Figure 12c), tiny grooves become the main feature and there are almost no flaking pits on it; this may be due to the softening of the contact surface at moderate temperatures. As for the PT sample (Figure 12d), the grooves become tiny and some delamination appears on the worn surface. A large extent of delamination with cracks and grooves is present on the sliding surface for PTP10 (Figure 12f). In summary, the main wear mechanisms are abrasive and fatigue wear at 100 °C.

SEM images of the sliding surface for PTFE matrix composites at 150 °C are presented in Figure 13. The worn surface becomes smooth and there are only some flaking pits across the contact surface, as compared with the case at 100 °C for PCTP (Figure 13c), PTP5 (Figure 13e) and PTP10 (Figure 13f) composites; this may be due to the plastic deformation of the PTFE matrix and the reinforcement of PEEK. As for PC (Figure 13a), PCT (Figure 13b) and PT (Figure 13d) composites, there are large grooves and severe plastic deformation, indicating that abrasive wear is the main wear mechanism.

As the testing temperature increases to 200 and 250 °C, we can see that the worn surface characteristics are similar to those at 50 °C, 100 °C or 150 °C for PC, PT, PTP5, PTP10 (the main features are grooves, as shown in Figure 14a,d–f and Figure 15a,d–f) and PCT composites (the main features are large grooves, delamination and cracks, as shown in Figure 14b and Figure 15b), indicating that the main wear mechanism is abrasive wear. For the PCTP composite, the worn surface is covered by a great extent of delamination, grooves and cracks at 200 °C (Figure 14c); as the testing temperature increases to 250 °C (Figure 15c), grooves and flaking pits become the main features, the above information suggesting that abrasive and fatigue wear are the main wear mechanisms at 200 °C and that abrasive wear is the main wear mechanism at 250 °C.

**Figure 16:**
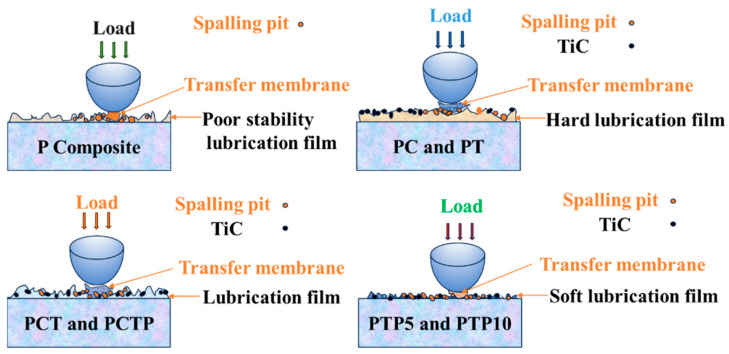
Schematic diagram of wear mechanism for different composites.

This comprehensive comparison of the friction coefficient and wear rate shows that the frictional behavior of PTFE matrix composites is quite dependent on the testing temperature and second particle content. To further understand the wear mechanism of the composites, a schematic diagram is drawn, as shown in Figure 16. For pure PTFE material, due to the easy slippage of the interlayer and plastic deformation, the highest wear rate is presented over the testing temperature. For PC and PT composites, the addition of CF and TiC could enhance the non-deformability of the contact surface and inhibit the interlayer slippage, especially at low to moderate temperatures, so these two composites exhibit relatively lower wear rates when compared with that of pure PTFE material. As PEEK is added or all of these three particles are added in the composite, we can see that the wear resistance property is further improved, especially for PCTP and PTP10; this can be attributed to the synergistic enhancement effect of CF, TiC and PEEK. As the PEEK molecular chain could adsorb PTFE matrix molecules and the TiC/CF phase could increase the hardness of the PTFE matrix and support in resisting the applied load, both PEEK and TiC/CF could inhibit the interface slippage of PTFE molecules; thus, the high wear resistance of PTFE matrix composites is exhibited, and this is consistent with the changing trend in wear rate (Figure 5).

## 4. Conclusions

In the present work, PTFE matrix composites with CF, TiC and PEEK were prepared by the cold pressing and hot sintering method, and the influence of second particles and testing temperature (from 25 to 250 °C) on tribological behavior was studied. The main conclusions can be summarized as follows:

(1) From 25 to 250 °C, the COFs for composites show a slight fluctuation (0.16–0.24). Due to the softening of the worn surface and the increased diffusion rate of PTFE and PEEK, four composites exhibited the minimum COF at 250 °C. By comparison, the PTCT composite shows the best lubricating performance over the testing temperature (0.16–0.17).
(2) All six PTFE matrix composites exhibit low wear rates when compared with pure PTFE material; the data decrease from (2.04–2.72) × 10^−3^ mm^3^/Nm for pure PTFE material to (0.674–10.6) × 10^−4^ mm^3^/Nm for other composites. Overall, the PCTP composite exhibits the best wear resistance properties, with a value below 1.96 × 10^−4^ mm^3^/Nm.
(3) From 25 to 250 °C, the effective lubricant and wear resistance performance is attributed to the synergistic effect of TiC/CF and PEEK. The main wear mechanisms are abrasive and fatigue wear at low-to-moderate temperatures (25–100 °C), and these change into abrasive wear from 150 to 250 °C.
(4) In future work, we plan to investigate the influence of extreme testing conditions on the friction and wear behavior for PTFE matrix composites, including ultralow temperatures (−50 °C, −100 °C and −150 °C). The results obtained in this work will provide theoretical guidance for the design and preparation of self-lubricating polymer matrix composites.

## Figures and Tables

**Table 1:** Composition of the PTFE matrix composites.

Materials	Composition	PTFE (wt. %)	CF (wt. %)	TiC (wt. %)	PEEK (wt. %)
PC	PTFE + CF	95	5	0	0
PCT	PTFE + CF + TiC	90	5	5	0
PCTP	PTFE + CF + TiC + PEEK	85	5	5	5
PT	PTFE + TiC	95	0	5	0
PTP5	PTFE + TiC + PEEK	90		5	5
PTP10	PTFE + TiC + PEEK	85	0	5	10

## Data Availability

Data are contained within the article.
